# Steroid Tumor Environment in Male and Female Mice Model of Canine and Human Inflammatory Breast Cancer

**DOI:** 10.1155/2016/8909878

**Published:** 2016-04-18

**Authors:** Sara Caceres, Laura Peña, Gema Silvan, Maria J. Illera, Wendy A. Woodward, James M. Reuben, Juan C. Illera

**Affiliations:** ^1^Department of Animal Physiology, School of Veterinary Medicine, Complutense University of Madrid (UCM), 28040 Madrid, Spain; ^2^Department of Animal Medicine, Surgery and Pathology, School of Veterinary Medicine, Complutense University of Madrid (UCM), 28040 Madrid, Spain; ^3^Department of Radiation Oncology, The University of Texas MD Anderson Cancer Center, Houston, TX 77030, USA; ^4^Department of Hematopathology, The University of Texas MD Anderson Cancer Center, Houston, TX 77030, USA

## Abstract

Canine inflammatory mammary cancer (IMC) shares clinical and histopathological characteristics with human inflammatory breast cancer (IBC) and has been proposed as a good model for studying the human disease. The aim of this study was to evaluate the capacity of female and male mice to reproduce IMC and IBC tumors and identify the hormonal tumor environment. To perform the study sixty 6–8-week-old male and female mice were inoculated subcutaneously with a suspension of 10^6^IPC-366 and SUM149 cells. Tumors and serum were collected and used for hormonal analysis. Results revealed that IPC-366 reproduced tumors in 90% of males inoculated after 2 weeks compared with 100% of females that reproduced tumor at the same time. SUM149 reproduced tumors in 40% of males instead of 80% of females that reproduced tumors after 4 weeks. Both cell lines produce distant metastasis in lungs being higher than the metastatic rates in females. EIA analysis revealed that male tumors had higher T and SO4E1 concentrations compared to female tumors. Serum steroid levels were lower than those found in tumors. In conclusion, IBC and IMC male mouse model is useful as a tool for IBC research and those circulating estrogens and intratumoral hormonal levels are crucial in the development and progression of tumors.

## 1. Introduction

Human inflammatory breast cancer (IBC) is the most aggressive mammary neoplasia that affects women [1, 2]. IBC accounts for less than 6% of human breast cancer diagnoses with the poorest survival in women [2, 3]. Canine inflammatory mammary cancer (IMC) has been proposed as the best spontaneous animal model for the study of human IBC [4]. The main histological characteristic of the disease in both species is the massive invasion of dermal lymphatic vessels by neoplastic cells which blocks lymph drainage causing the characteristic edema [5, 6]. In both species, this type of cancer is highly angiogenic and angioinvasive [4, 7–9].

Several human IBC cell lines have been established in order to study the mechanisms of this special type of breast cancer* in vitro *such as SUM149, SUM190, and MDA-IBC-3 [10–12]. Recently IPC-366, an IMC triple negative cell line, has been established [13].

On the other hand, male breast cancer (MBC) is a rare disease that accounts for less than 1% of all breast carcinomas [14] and male inflammatory breast cancer is extremely rare [15]; however, the incidence of MBC is increasing [16]. MBC appears to be biologically similar to female breast cancer [16] and it has been found that clinical and histological features in male and female inflammatory breast cancer are also similar [15]. Hormone imbalance between estrogens and androgens levels is one of the main risk factors for MBC [14], as it is known that androgens exert inhibitory effects in hormone-dependent breast cancer cells [14, 17], but the role of androgens in breast cancer development is still unclear.

Several studies have demonstrated that the tumor tissues have a local steroid synthesis [18]. Biologically active estrogens are locally produced by estrogen-producing enzymes, such as aromatase that converts circulating androstenedione to estrone or testosterone to estradiol in breast carcinoma [19]. Also intratumoral androgens concentrations were reported to be significantly higher in breast carcinoma [20], and androgen-producing enzymes, such as 17bHSD5 that converts circulating androstenedione to testosterone, and 5a-reductase type 1, which reduces testosterone to DHT, were expressed [21].

The development of xenografts has been a useful tool for improving our understanding of breast cancer progression and metastasis [22–24]. The majority of breast cancer xenografts are performed on female mice. Therefore, the aim of this study was to conduct a male mouse model of IBC and IMC to determine the similarities and differences between female and male tumor progression and tumor hormonal environment.

## 2. Materials and Methods

### 2.1. Cell Culture

Canine inflammatory mammary carcinoma cell line, IPC-366, was cultured in Dulbecco's Modified Eagle Medium Nutrient Mixture F-12 Ham (DMEM/F12) containing 10% fetal bovine serum, 1% L-glutamine, and 1% antibiotic-antimycotic (Sigma Aldrich). Its human counterpart, SUM149 cell line, was obtained from Asterand plc (Detroit, MI) and was cultured in Ham's F12 (Fisher Scientific) supplemented with 10% fetal bovine serum, 5 *μ*g/mL insulin, 1 *μ*g/mL hydrocortisone, and antibiotic-antimycotic (Sigma Aldrich).

All cell lines were cultured in 25 cm^2^ culture flasks and were maintained in a humidified atmosphere of 5% carbon dioxide at 37°C. Cell culture was observed daily by a phase-contrast microscopy.

### 2.2. Animals

A total of sixty 6–8-week-old female (F) mice and male (M) mice BALB/cJHan®Hsd-Prkdcscid (SCID) (Harlan Laboratories Models, SL, Barcelona, Spain) were used in this study, divided into the following groups: 20 (10 F and 10 M) as serum control group and 40 (F and M) inoculated with IPC-366 (*n* = 20) and SUM149 (*n* = 20) as experimental groups (serum and tumor homogenates). The animals were housed in a flexible-film isolator (Isotec, Harlan Laboratories Models, SL) in cages (1-2 animals per cage), in a room with controlled environmental conditions (20–22°C; 50–55% relative humidity; 10–15 air changes per hour; and a 12 : 12-hour light : dark cycle). Food and water, previously sterilized, were provided ad libitum. Prior to all procedures, animals were anesthetized with isoflurane (IsoVet) at 4% for induction and 1.5% for maintaining sedation, supplied in a fresh gas flow rate of 0.5 L of oxygen/minute, and were observed until fully recovered. Animals were sacrificed by a lethal dose of isoflurane.

Clinical and experimental protocols of this study were approved by the Institutional Animal Care and Use Committee of the University Complutense of Madrid, Spain (number: 115). All procedures were completed in accordance with the Guide for the Care and Use of Laboratory Animals and conformed to the relevant EU Directive.

### 2.3. Mice Cell Inoculation

A suspension of 10^6^ IPC-366 cells and 10^6^ SUM149 cells were implanted subcutaneously into the fourth inguinal mammary gland. Mice were inspected twice/week for the development of tumors. If tumors were detected, they were weekly monitored by palpation and measured by calipers. Mice were sacrificed when tumor volume was up to 1500 mm^3^. Blood samples were taken from the submandibular venous sinus. Tumors were collected at necropsy for homogenates.

### 2.4. Steroid Determinations in Serum and Tumor Homogenates

Tumors were homogenized in 4 mL of PBS (pH 7.2) and centrifuged at 1200 g, for 20 min at 4°C. Supernatants were collected and aliquoted individually (−80°C) until hormone assays. Blood samples were centrifuged at 1200 g and 4°C for 20 min and serum was separated and stored frozen at −20°C until assayed. Estrone sulphate (SO4E1: ab R522-2), 17b-estradiol (E2: ab C6E91), androstenedione (A4: ab C9111), testosterone (T: R156), and progesterone (P4: C914) levels of tumor homogenates and serum samples were assayed by enzyme-immunoassay (EIA) previously validated [25]. All antibodies were developed in the Department of Animal Physiology (UCM, Spain).

All hormone concentrations were expressed in ng/g (for tumor homogenates) and ng/mL (for serum samples), except serum E2 concentrations that were expressed in pg/mL.

### 2.5. Statistics

The statistics software used for data analysis was SAS 9.4 (UCM, Madrid, Spain). The results were expressed as the means ± SD. For tumor progression analysis, to compare both cell lines (IPC-366 and SUM149) in each group the Wilcoxon rank-sum test was performed. For comparisons between groups on each cell line, we used the Kruskal-Wallis test followed by a pairwise nonparametric multiple comparisons test when the overall contrast was significant. Wilcoxon signed ranks test with Bonferroni correction was used for comparisons between weeks on each group and cell line. Differences in hormonal concentrations between group means were analyzed by one-way analysis of variance (ANOVA) followed by appropriate* post hoc* tests for similar variances (Duncan Test) or different ones (Games Howell test). In all statistical comparisons, *p* < 0.05 was accepted as denoting significant differences.

## 3. Results

### 3.1. Tumor Growth Progression in Male and Female Mice

IPC-366 and SUM149 cells were injected subcutaneously on female and male SCID mice to observe if there were differences in tumor growth parameters (Table 1). All female mice inoculated with IPC-366 cells reproduced a tumor that was appreciable approximately two weeks after cell injection (16.64 ± 1.72 days). However, 80% of female mice inoculated with SUM149 cells reproduced a tumor with significant difference (*p* < 0.05) in tumor appearance that was in approximately 4 weeks after cell injection (26.82 ± 2.19 days).

Male mice showed similar results on each cell line. Results revealed that 90% and 40% of male mice inoculated with IPC-366 and SUM149, respectively, originated tumors. In SUM149, frequency of tumor appearance in males was halved with respect to females, being a significant difference (*p* < 0.05). IPC-366 and SUM149 males showed no statistically significant differences with respect to females in time of tumor occurrence and time in which tumor volume of 1500 mm^3^ was reached.

Results of frequency of mice that developed ulceration and metastasis also differed between males and females inoculated with both cell lines. The percentage of ulcerations and metastasis found in males was reduced in contrast to the females. Frequency of ulceration was significantly higher (*p* < 0.05) in females (IPC-366 50%; SUM149 30%) than in males (IPC-366 10%; SUM149 0%). Also, both models developed spontaneous distant metastasis with significantly higher (*p* < 0.05) frequency in females (IPC-366 90%; SUM149 80%) than males (IPC-366 20%; SUM149 50%).

**Figure 1:**
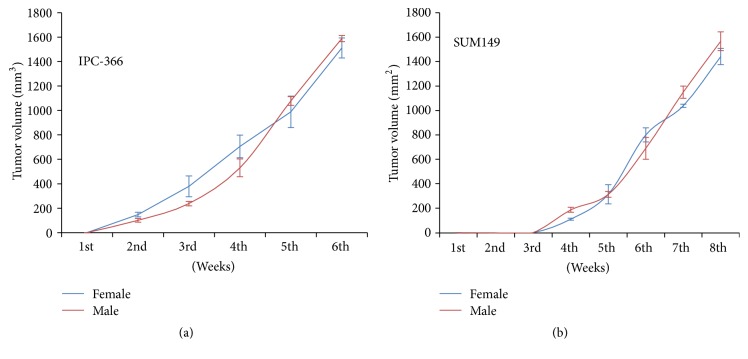
*In vivo* tumor growth progression of (a) IPC-366 and (b) SUM149 male and female mice. Tumor growth followed the same pattern in males and females in both cell lines. Lines represent means ± SD. There were no statistical differences between groups.

Tumor growth progression in males and females of IPC-366 and SUM149 cell lines followed a similar pattern (Figure 1). Both models exhibited rapid growth* in vivo* reaching a volume of 1500 mm^3^ approximately 6–8 weeks after cell inoculation and no statistical differences were found in tumor progression results.

### 3.2. Hormonal Tumor Environment

**Figure 2:**
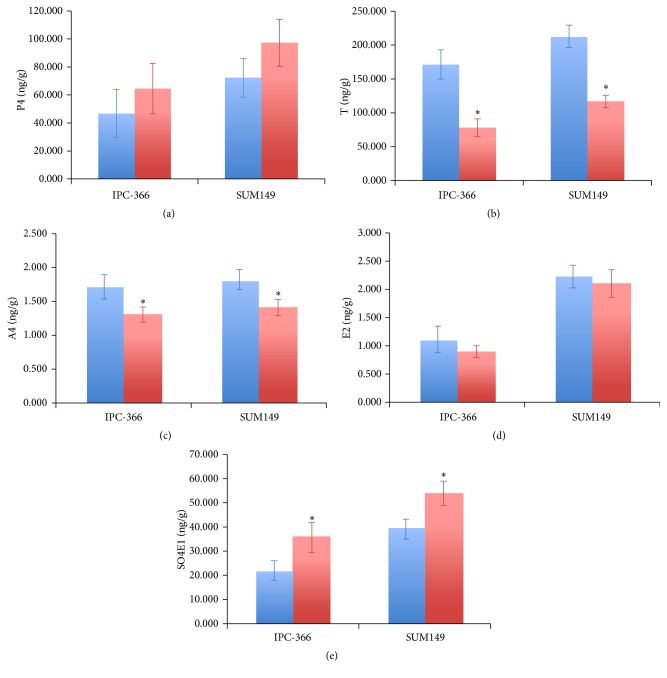
Hormonal levels of (a) P4, (b) T, (c) A4, (d) E2, and (e) SO4E1 of male and female IPC-366 and SUM149 tumors homogenates. Estrogens were higher in female tumors than in males instead of androgens that were higher in males tumors than females tumors. Bar represents means ± SD. ^*∗*^ *p* < 0.05 denoted significant differences between females and males.

Steroid determinations in tumor homogenates revealed that tumors from males and females differed on estrogen and androgen levels (Figure 2). P4 levels were lower in males compared with female P4 levels but not significantly. However, A4 and T levels were significantly higher (*p* < 0.05) in males than in females. Estrogen levels were higher in female tumors than in males with this difference being significant (*p* < 0.05) in SO4E1 levels, but E2 levels did not show any significant difference.

### 3.3. Serum Hormonal Concentrations

Results from serum steroid concentrations (Table 2) in control and experimental groups showed that control mice had significantly higher (*p* < 0.05) steroid levels than IPC-366 and SUM149, except A4 concentrations that were significantly higher (*p* < 0.05) in IPC-366 and SUM149 mice than in control mice. Differences between females and males in control and experimental mice were also found. SO4E1, E2, and P4 were significantly higher (*p* < 0.05) in female mice than in males. SUM149 mice did not show any statistical differences in these steroid concentrations. However, IPC-366 mice showed statistical differences (*p* < 0.05) in E2 and P4 levels but not in SO4E1, being higher in females than males. Besides, T levels were significantly higher (*p* < 0.05) in males than females in control, IPC-366, and SUM149 mice, but in A4 concentrations any statistical difference between females and males was found.

## 4. Discussion

IMC and IBC are considered the most malignant and aggressive subtypes of breast cancer that affect female dogs and humans, respectively, [3–5] and IMC has been suggested as a model to study the human disease [4, 5]. Recently, a triple negative IMC cell line (IPC-366) has been established as a useful tool for breast cancer research [13].

Animal models are of great value in cancer research. However, the microenvironment around the tumor is crucial for tumor development [26] and hormonal secretion plays an important role.

This study was intended to develop a male animal model for elucidating which endocrine factors may be involved in breast carcinogenesis, comparing tumor growth and intratumoral steroids levels in female and male mice inoculated with IBC and IMC cell lines (SUM149 and IPC-366 cell lines, resp.).

Our results revealed that both cell lines were capable of reproducing tumors in male mice at the same time compared to female mice, but the frequency of tumor appearance was lower than in female mice. The reason of the lower frequency rates found in male mice could be due to the androgen environment that male mice provide as it is known that androgens exert inhibitory effects in hormone-dependent breast cancer cells [17]. To our knowledge, this is the first male animal model for breast cancer research, as male breast cancer development is still unclear. We also found that metastases and ulceration rates were also lower in males. Tumor metastasis comprises different processes that lead tumor cells move away from the tumor to a distant location [27] and some authors suggested that stromal cells regulate the production of various factors implicated in metastasis process such as COX2, TNF-*α*, IL-6, and IL-11 [28]. However, hormone levels could also exert an influence on metastatic process. In point of fact, several authors' associate levels of expression of SO4E1 with lymph node metastases [29] that is also in agreement with our results in female mice, where we found higher amount levels of intratumoral SO4E1 and metastatic rates.

In ER positive breast carcinomas, androgens are well known to suppress cell proliferation but there is poor knowledge of the roles of androgens in triple negative carcinomas [30]. It is known that ER/PR negative carcinomas are associated with decreased hormone levels of androgens and estrogens when compared to ER/PR positive cancers [18]; this hypothesis was supported by Blankenstein et al. that observed significant estradiol levels in ER-negative tumors [31].

Wiebe suggested that P4 metabolites produced within breast tissues might function as cancer promoting or inhibiting agents, since P4 serves as the precursor for the major steroid hormones (androgens and estrogens). Tumor progression could be related to changes in local P4 levels [32]. Our results reveled that intratumoral P4 levels were decreased in male mice compared to those found in females. The decrease of intratumoral levels of P4 in males compared to females might be due to the low frequency of metastasis found in males, as it is proposed that P4 metabolites might play a role in the acquisition of metastatic potential [32].

Estrogens are known to be responsible for development and progression of breast cancer by stimulating cell proliferation [33]. E2 is the most potent estrogen whose effects are mediated by binding to the estrogen receptor (ER) [27, 34]. Likewise, it is known that androgens suppress cell proliferation in breast cancer cells [17]; however, their role in carcinogenesis on breast tissue is still unclear and there is some controversy on their effects on breast cancer [35]. Some studies revealed that androgens mediated cell growth via aromatization in epithelial breast cancer cells [36].

*In situ* production of steroids plays an important role on steroid signaling in hormone-dependent carcinomas. These tumors do not depend on circulating steroid levels but produce steroid hormones locally from circulating precursors [37]. Additionally, the local synthesis of steroids has been proposed in the canine mammary gland [38] and the ability of canine and human breast cancer cell lines to produce steroid hormones in* in vitro* conditions [39].

Serum hormone levels were lower than those found in control group, suggesting that estrogens and androgens locally produced in tissues act without being released into the bloodstream [20]. These results provide evidence that tumoral tissue uptake plasma steroid from circulation and also produce the biosynthesis of them [31, 38]. Estrogens tissue levels will be the result of biosynthesis and degradation of the estrogenic enzymes. Apart from the enzyme activity, estrogen biosynthesis will also depend on the availability of substrate. The breast does not have the precursor steroids such as androstenediol or testosterone, which directly convert to E2, DHEA, and A4, and can contribute to E2 biosynthesis via estrone [31].

In this study we found several differences in intratumoral estrogen and androgen levels between females and males. Males had higher levels of intratumoral androgens instead of females that presented higher levels of estrogens. Our study confirms the hypothesis of estrogen local production in IMC and IBC [38, 40, 41] because higher SO4E1 and E2 concentrations were found in tumor than in serum. As androgens suppress cell proliferation, the high intratumoral androgen levels found in males could be associated with the low frequency of tumors in males inoculated with SUM149 and also the low metastases rates in both cell lines. Probably, intratumoral androgens exert an effect on the stromal cells by blocking the metastatic process in male mice. Planas-Silva and Waltz found that E2 promotes reversible epithelial-to-mesenchymal transition in ER*α*-positive cells [42] and these changes in the cells could lead to metastasis [27]. Thus, the high estrogen intratumoral levels found in female mice could also be implicated on the metastatic process and the malignancy of the cells. We have found that in females SO4E1 intratumoral levels were significantly higher than in males, and E2 levels were similar in both models. Probably, the amounts of SO4E1 found in females could act as a reservoir of estrogens [43]. In the case of males, to counteract high T intratumoral levels and promote tumor progression, tumor cells use SO4E1 reservoirs to produce biologically active estrogens (E2) and thus promote cell proliferation.

## 5. Conclusions

In this study we determined an IBC and IMC male mouse model useful for male and women inflammatory breast cancer. We also have found that hormonal tumor environment is crucial for tumor development and progression; high amounts of intratumoral androgens could be associated with a low risk of metastatic capacity.

## Figures and Tables

**Table 1:** Tumor growth parameters of female and male mice inoculated with IPC-366 and SUM149 cells.

Cell line	Gender	% of animals with tumor	Time of palpable tumor (days)	Time of 1.500 mm^3^ volume (days)	% of animals with ulceration	% of animals with metastasis
IPC-366 (*n* = 20)	Female	100%	16.64 ± 1.72	42.02 ± 2.35	50%	90%
	Male	90%	15.16 ± 2.60	39.66 ± 3.29	0%^*∗*^	20%^*∗*^

SUM149 (*n* = 20)	Female	80%	26.82 ± 2.19^a^	53.40 ± 4.86^a^	30%	80%
	Male	40%^*∗*,a^	24.50 ± 3.5^a^	51.33 ± 3.66^a^	10%	50%^*∗*,a^

^*∗*^ *p* < 0.05, significant differences between females and males inoculated with each cell line. ^a^Significant differences (*p* < 0.05) between cell lines.

**Table 2:** Serum steroid concentrations in female (F) and male (M) mice in control group, inoculated with IPC-366 and SUM149 cells.

Steroid hormone	Gender	Control	IPC-366	SUM149
SO4E1 (ng/mL)	F	1.30 ± 0.03^a^	0.11 ± 0.12^b,1^	0.12 ± 0.01^b,1^
	M	0.29 ± 0.04^*∗*,a^	0.07 ± 0.02^b,1^	0.09 ± 0.03^b,1^

E2 (pg/mL)	F	42.79 ± 3.64^a^	6.73 ± 0.37^b,1^	8.67 ± 0.71^b,1^
	M	6.00 ± 0.26^*∗*,a^	2.11 ± 0.12^*∗*,b,1^	2.91 ± 0.22^*∗*,b,1^

A4 (ng/mL)	F	0.21 ± 0.03^a^	0.64 ± 0.13^b,1^	0.78 ± 0.18^b,1^
	M	0.14 ± 0.02^a^	0.48 ± 0.09^b,1^	0.56 ± 0.11^b,1^

T (ng/mL)	F	0.5 ± 0.01^a^	0.31 ± 0.09^a,1^	0.45 ± 0.12^a,1^
	M	2.3 ± 0.6^*∗*,a^	1.66 ± 0.39^*∗*,a,1^	1.72 ± 0.28^*∗*,a,1^

P4 (ng/mL)	F	3.59 ± 0.04^a^	0.48 ± 0.22^b,1^	0.57 ± 0.07^b,1^
	M	1.10 ± 0.32^*∗*,a^	0.18 ± 0.03^*∗*,b,1^	0.32 ± 0.05^b,1^

^*∗*^ *p* < 0.05, significant differences between females and males. Different letters denoted statistical differences (*p* < 0.05) between control and cell lines. Different numbers denoted statistical differences (*p* < 0.05) between cell lines.
